# Development and simulation of a novel mathematical model for an intelligent tire system toward predictive maintenance

**DOI:** 10.1038/s41598-026-38625-1

**Published:** 2026-02-09

**Authors:** Hassan Hijry, Saeed Mohsen, Omar Albalawi, Saad Ali, Ali Alhawiti, Gamal A. Elnashar

**Affiliations:** 1 Department of Industrial Engineering, Faculty of Engineering, University of Tabuk, 47512 Tabuk, Saudi Arabia; 2 Department of Electronics and Communications Engineering, Al-Madinah Higher Institute for Engineering and Technology, Giza, 12947 Egypt; 3 Civil Engineering Department, Faculty of Engineering, University of Tabuk, 71491 Tabuk, Saudi Arabia; 4 Department of Artificial Intelligence Engineering, Faculty of Computer Science and Engineering, King Salman International University (KSIU), South Sinai, 46511 Egypt

**Keywords:** Smart tire, Flexible ring model, Tire physical model, Tire wear, Strain signal, Engineering, Materials science, Mathematics and computing

## Abstract

An intelligent tire system autonomously collects and transmits key data on tire and road conditions. As vehicle performance depends on tire dynamics governed by tire–road interactions, accurate tire modeling is essential for optimizing overall performance. Tire deformations significantly influence longitudinal and lateral forces, emphasizing the need for accurate predictive models. The growth of autonomous vehicles (AVs), electric vehicles (EVs), and shared mobility has intensified research on tire–vehicle interactions. EVs, in particular, place special demands on tires due to their heavier battery packs, immediate torque output, and regenerative braking, which can increase tire wear by 20–30% compared to traditional vehicles. Smart tire models have been widely explored to improve vehicle control systems, as embedded sensors provide valuable real-time data on tire behavior under actual driving conditions. These models seek to link measured parameters with physical states to improve prediction accuracy. This paper introduces a physical-analytical approach to describe in-plane tire dynamics by connecting deformations to the forces involved. Tire parameters are determined by aligning radial deformation data from a flexible ring model with strain-based simulations. The proposed mathematical model, based on carcass strain and displacement data, provides a solid theoretical foundation for future intelligent tire research and aids further studies on tire wear, lifespan, mechanical performance, and predictive maintenance. This work offers a theoretical foundation for intelligent tire systems, solutions that significantly reduce computational effort compared to numerical methods, and a potential direct indicator for wear estimation algorithms.

## Introduction

Tire modelling is an essential method for depicting the mechanical, thermal, and wear properties of tires. These models usually involve mathematical equations that relate input factors, like loads or slip, to the tire’s responses. They form the basis for various applications, including vehicle dynamics analysis, advanced control techniques, ride comfort evaluation, and energy efficiency enhancements. However, no single tire model fits all scenarios due to the diverse requirements in the automotive and tire industries. To meet these different needs, a wide range of tire models has been developed, from simple to highly complex. One well-known simplified model is the brush model, which, despite its simplicity, effectively captures key elements of tire dynamics. This model represents the tread as a set of flexible bristles, illustrating deformation and overall movement within the contact patch. This approach helps explain how forces and moments arise at the tire–road interface and how frictional limits affect their formation. Fiala^1^ proposed dimensionless formulas based on the brush model to mathematically describe lateral force generation and aligning moment behaviour of tires. Due to its relatively few parameters, this model is easy to implement, but its accuracy is limited since it does not consider changes in cornering stiffness or friction coefficient variations. Additionally, reference^2^ offers a thorough review of current technologies and estimation methods related to intelligent tire systems.

Much research on intelligent tire systems uses sensors attached to the tire’s inner liner, mainly categorized as acceleration-based or strain-based sensors^3^. Beyond these common methods, other sensor technologies have shown promising results. For instance, Magori et al.^4^. developed an ultrasonic sensor mounted on the wheel rim to monitor tire deformation, contact patch, and temperature characteristics. Similarly, Tuononen has extensively applied optical sensing to track carcass deflection, allowing direct estimation of vehicle state parameters^5^. Moreover, researchers have created segmented capacitance ring sensors for real-time tire inflation pressure measurement^6^. The mathematical tire model presented improves predictions of carcass structural behavior under different driving conditions. Adapted from Gong’s foundational tire modelling work^7^, this model provides analytical solutions for radial and tangential tread displacements in real time, based on a simplified assumption of a parabolic normal pressure distribution at the contact area. These closed-form solutions significantly reduce computational effort and enable qualitative estimation of tire forces when lateral and longitudinal slip occur simultaneously. Intelligent tire technology typically depends on sensor arrays that respond to deformations within the contact patch. Over time, various sensing methods—such as triaxial acceleration sensors^8,9^, strain gauges^10^, and PVDF piezoelectric films^11^—have gained popularity due to their resistance to interference and ease of installation. These sensors mainly measure acceleration, strain, or deformation, which are essential for estimating tire-road interaction parameters like contact patch size, road texture, friction coefficient, tire load, slip ratios, and slip angles. Unlike sensors mounted on the vehicle chassis, those embedded in the tire directly capture the detailed mechanical interactions at the tire-road interface, providing more comprehensive real-time data on contact conditions. As a result, intelligent tire systems mark a significant advancement in enhancing vehicle safety and control capabilities.

Tire manufacturers will need to navigate legislation concerning safety, CO_2_ emissions (related to rolling resistance), and noise (external). It will be essential for future tire models to effectively address these challenges and provide precise estimations of tire performance in such scenarios.

- The Magic Formula tire model, created by Hans B. Pacejka, is a semi-empirical framework designed to replicate the forces and moments produced by a tire through a set of equations that utilize curve-fitting constants. A significant drawback of this model is its potential instability at very low speeds, which can lead to divergence. The model’s performance is highly dependent on its parameters, making the accurate determination of these coefficients vital.
- The Brush model is a physically grounded approach that offers valuable insights into the mechanisms occurring within the contact patch. It served as the foundation for early investigations into phenomena such as tire shimmy. Subsequent iterations of the brush model have been developed to include additional parameters, enhancing their accuracy.
- Contemporary methodologies often merge the advantages of various models. For instance, certain brush-type models are augmented with extra parameters that are calibrated using real-world data to enhance their precision, effectively bridging the divide between a purely physical and an empirical methodology.

In literature, previous research focused on a Tire Pressure Monitoring System (TPMS), which is an electronic safety feature in vehicles that alerts the driver when one or more tires are significantly under-inflated. It is mandatory on all new passenger vehicles sold in the U.S. since 2008 and in the EU since 2014. Although TPMS was a great milestone, the proposed intelligent tire idea has bigger prospects than just TPMS. Researchers focusing on smart tire technologies leverage these sensors in combination with sophisticated estimation methods, ranging from analytical tire models to neural network algorithms, to quantify tire-road forces and moments effectively. Within the intelligent tire system, sensors represent the cornerstone component; their precision and reliability critically determine the overall accuracy and robustness of the system’s perception capabilities regarding tire-road dynamics. To provide detailed data for validating and parameterizing the proposed flexible ring tire model (FRTMs) for research and simulation, FRTM-compatible sensors are used, specialized sensors, like strain gauges, accelerometers, or optical sensors measure specific aspects of tire behaviour, such as deformation, vibration, or displacement.

The main goal of this paper is to develop and simulate a novel physical–analytical mathematical model for an intelligent tire system that accurately describes in-plane tire dynamics by linking tire deformations with the generated forces and moments at the tire–road interface. The model—based on the FRTM—aims to provide closed-form analytical solutions for tire displacement and strain, enabling realistic, low-computation simulations that can predict tire behavior under different loads, pressures, and slip conditions. A new coupled rigid-flexible ring model has been introduced to examine the dynamic response characteristics of tires. The contact algorithm, which is based on a 2D flexible ring, facilitates the analysis of pressure distribution within the tire-road contact area under varying vertical loads.

The key contributions of this paper are the following:

- A new coupled rigid-flexible ring model has been introduced to examine the dynamic response characteristics of tires. The contact algorithm, which is based on a 2D flexible ring, facilitates the analysis of pressure distribution within the tire-road contact area under varying vertical loads.
- This study proposes a physical model that incorporates tire wear. The model is an improvement over the traditional flexible ring model, incorporating brush theory.
- Closed-form solutions for displacement and strain: the model derives analytical expressions for radial and tangential tread displacements and circumferential strain under steady-state conditions.
- Discovery of wear-dependent strain signatures: identifies a distinctive strain response pattern at the trailing edge of the contact patch as tire wear progresses.
- A strain-based theoretical foundation for estimating tire-road interaction parameters (e.g., forces, friction) using embedded sensors, enhancing vehicle safety and control without complex pre-processing is established.

The organization of this paper is as follows: “Theoretical background concerning intelligent tires withembedded sensors” offers an in-depth overview of the tire sensor technologies used. “The proposed mathematical modelling of tire system” details the proposed modelling approach, while “Simulation results of smart tire strain behaviour” presents results and analyzes the related simulation outcomes. Lastly, “Conclusions and future work” concludes the main findings and suggests possible avenues for future research.

## Theoretical background concerning intelligent tires with embedded sensors

**Fig. 1:**
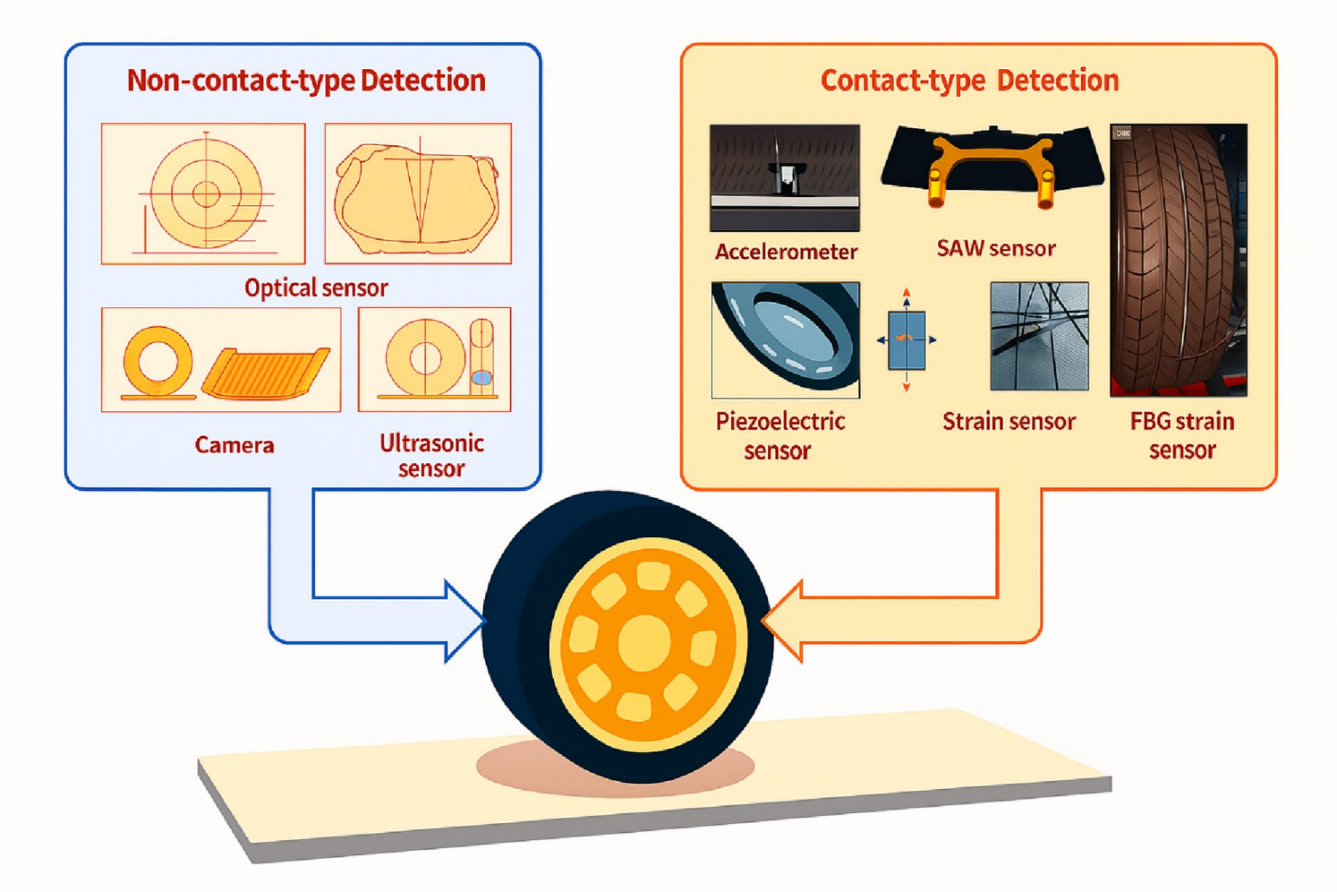
Intelligent tires with embedded sensors.

Smart tires integrate advanced sensing technologies within their structure, enabling continuous monitoring of numerous tire and road conditions. These built-in sensors provide real-time data aimed at improving vehicle safety and driving performance by tracking parameters like tire pressure, temperature, road surface, and tread wear features like roughness and friction levels. Based on their location and operating principle, these sensors are generally categorized as either contact or non-contact types, as shown in Fig. 1^12^. Contact sensors are usually installed on the tire’s inner surface, allowing them to deliver direct and immediate measurements of tire conditions. Conversely, non-contact sensors, such as those using optical or ultrasonic methods, gather tire state information without physically touching the tire. Tire sensors are advancing from traditional Tire Pressure Monitoring Systems (TPMS) to “intelligent tires” that deliver real-time, high-accuracy information regarding tire and road conditions. These sensors hold significant engineering potential for enhancing vehicle control, increasing safety, and supplying real-time data that benefits both driver awareness and sophisticated system functionalities such as stability and traction control. They are capable of monitoring tire pressure, temperature, slip angle, and the road friction coefficient, which facilitates more accurate adjustments to driving conditions and enhances vehicle stability.

### Contact-type detection sensors

#### Accelerometers

References^13,14^ describe the application of one or more accelerometers attached to the tire’s inner liner or sidewall to estimate essential parameters such as slip angle, tire forces, inflation pressure, and friction properties, and to distinguish between different types of road surfaces. Typically, triaxial acceleration sensors are used, which are usually bonded to the inner tire surface with a silylated urethane adhesive to ensure secure attachment during dynamic conditions. The placement of these sensors can vary widely, ranging from symmetrical or parallel arrangements along the tire’s lateral axis to asymmetrical layouts, and sometimes a single sensor is sufficient. Using multiple sensors distributed laterally allows researchers to examine variations across the contact patch width, providing deeper understanding of tire behavior under sideslip and camber angle conditions.

#### Piezoelectric sensors

Piezoelectric strain sensors can be embedded within tires to monitor critical parameters, for instance, temperature, pressure, and wear, thereby improving the functionality of intelligent tire systems. Since tires experience cyclic deformation at specific points during rolling, sensors based on the piezoelectric effect are especially suitable for detecting these dynamic strain changes^15,16^. Usually made from materials like polyvinylidene fluoride (PVDF), these sensors convert mechanical strain into electrical energy, enabling real-time deformation monitoring and potentially serving as a sustainable power source for the sensors or other embedded devices, which may reduce reliance on batteries. For example, in^17^, eight piezoelectric sensors were strategically placed at four different positions on the tire, with two sensors at each location oriented longitudinally and radially to effectively capture multidirectional deformation signals.

#### Strain gauges

Strain gauges have been embedded in the tire’s inner liner^18^ to directly measure tire operating conditions under both static and dynamic scenarios. As noted in^19^, these sensors have been used to track important parameters such as effective rolling radius, contact patch length, and instantaneous velocity at the contact-patch. In this setup, three rectangular rosette strain gauges are attached to the tire in both lateral and longitudinal directions to gather multidirectional strain data. Although strain-gauge-based tire sensors, commonly used in Tire Pressure Monitoring Systems (TPMS), provide valuable information, they have several inherent drawbacks. These include sensitivity to temperature-induced measurement drift, susceptibility to mechanical damage or air leaks, and difficulties in maintaining accuracy over extended use. Additionally, their replacement can be costly, and servicing often requires specialized tools and expertise.

#### Electric conductive rubber sensors

The Pressure Sensitive and Electrically Conductive Rubber (PSECR) sensor is made from a unique rubber material whose electrical conductivity rises when compressed. This property makes PSECR sensors especially useful for measuring force and distributed pressure. They are appreciated for their flexibility, durability, and high sensitivity to both static and dynamic loads. As shown in^20^, PSECR sensors have been utilized to directly measure localized friction forces caused by tire–road stick–slip interactions. Additionally, according to^21^, these sensors can detect multidimensional compressive forces; the collected data are then incorporated into a tire model based on a beam spring approach, enabling the estimation of complex, multidimensional loading and friction parameters important for vehicle dynamics.

#### Surface acoustic wave sensors (SAW)

Surface Acoustic Wave (SAW) sensors function by utilizing the propagation of acoustic waves along the surface of a piezoelectric material to measure variables such as tire pressure, temperature, acceleration, and strain. These sensors offer several benefits: they are small and lightweight, highly resistant to environmental disturbances, making them suitable for precise measurements in harsh conditions, and they operate without needing an on-board battery. As described in^22^, SAW sensors are passive devices activated by a radio frequency signal. When excited, they generate a surface acoustic wave whose response can be received and analysed to obtain displacement-related data. By reflecting incoming radio signals from a processing terminal back into the air without requiring additional power, they enable wireless communication. Typically, SAW sensors use metallic interdigital transducers (IDTs) patterned on a piezoelectric substrate to create the acoustic wave, which is then converted back into an electrical signal by the same device attached to the tire. Changes in amplitude, frequency, or time delay between transmitted and received signals can be measured to determine tire operating conditions. Zhang et al.^23^. demonstrated the use of SAW sensors embedded in tires for simultaneous monitoring of pressure and temperature, showcasing their potential for multifunctional tire condition monitoring in intelligent vehicle systems^22^.

#### Fiber Bragg grating sensors (FBG)

FBG strain sensors have been utilized inside tires to monitor different operational conditions. These sensors originate from standard optical fiber technology commonly used in telecommunications. An optical fiber consists of three concentric layers: the central core with a relatively high refractive index; a surrounding cladding layer with a slightly lower refractive index that keeps light confined within the core; and an outer protective coating that provides mechanical strength. An FBG is a short grating inscribed within the fiber core that reflects specific wavelengths while allowing others to pass through. By examining phase changes in the reflected laser signal, precise measurements of surface displacement and strain in the material can be obtained. Research^24–26^ has shown the integration of FBG strain sensors into tires for condition monitoring, emphasizing their benefits such as being lightweight, immune to electromagnetic interference, and capable of detecting very high strain levels. However, despite these technical advantages, the use of FBG systems in smart tire applications is currently impractical due to their high cost, which reportedly surpasses that of luxury sports cars, making them unsuitable for widespread commercial use in intelligent tires.

### Non-contact-type detection sensors

Non-contact sensors are designed to measure parameters like position, displacement, distance, or other physical properties without direct physical contact with the target surface. This category includes various sensing technologies such as laser displacement, ultrasonic, capacitive, inductive, and magnetic sensors. These methods are widely applied in areas ranging from liquid level detection to object recognition and precise position measurement. In tire monitoring, non-contact sensors can evaluate deformation without touching the tread surface, thus avoiding common problems like debonding that can occur with contact-based sensors. Among the most commonly used non-contact solutions for tire condition monitoring are optical and ultrasonic sensors, both providing reliable and accurate measurements under operational conditions.

#### Optical sensors

Optical sensing technologies for tire monitoring generally fall into two main types. The first uses charge-coupled device (CCD) cameras to capture high-resolution images that are processed into continuous video streams. In these applications, the observed surface is often coated with carefully designed reference patterns, such as phase-shifting Moiré patterns, to enable precise measurement and deformation analysis^27^. The second type involves laser-based methods, particularly those employing FBG technology. Although both approaches offer high accuracy, their adoption is limited by high costs. Additionally, there is a lack of extensive research assessing the performance and durability of these optical sensing systems under challenging or harsh operating conditions.

#### Cameras and ultrasonic sensors

Cameras are another type of optical sensing device notable for their wide measurement capabilities. For example, in^28^, a CCD camera is installed inside the tire cavity and fixed to the wheel rim to track tire deformation. This setup allows for measuring in-plane strain, out-of-plane displacement, and internal tire pressure as part of the proposed sensing approach. Besides optical methods, ultrasonic sensing technology can also be used to evaluate tire conditions. In^29^, ultrasonic transducers are attached to the rim to measure the distance to the tread surface and the gap between the rim and the tire’s inner liner. After calibration, these measurements enable calculation of parameters such as two-dimensional contact sector area, contact-length, and the deformed tire profile. Generally, non-contact sensors provide high measurement accuracy but do not directly capture the interactions at the tire–road interface. Additionally, the hardware required for these systems tends to be complex and expensive, which limits their feasibility for real-world vehicle applications.

An effective smart tire sensor must meet several important design and operational criteria to ensure long-term reliability and practicality:

- Mechanical flexibility: The sensor should be flexible enough to accurately detect strain on the tire’s rubber surface, which has a relatively low Young’s modulus.
- Low mass impact: Its weight must be minimal to avoid disturbing the dynamic balance of the rotating wheel, as excessive mass can adversely affect wheel dynamics.
- Passive operation: Passive sensing methods are preferred because they simplify integration into challenging environments by removing the need for an independent power source.
- Protective coating: The sensing element should be properly encapsulated to prevent deterioration of its electrical properties while allowing secure attachment to the tire wall using an appropriate epoxy adhesive.
- Cost efficiency: The sensor must be affordable to enable mass production and widespread market adoption.
- Durability: It should be mechanically and functionally robust enough to last the typical service life of a mid-size passenger vehicle tire—about two years or 100,000 km—without losing performance.

## The proposed mathematical modelling of tire system

The main goal of an intelligent tire system is to convert the physical signals produced at the tire tread into significant engineering parameters, such as tire forces and the tire–road friction coefficient. To achieve this, a fundamental understanding of tire behaviour at the physical level is essential, whether the estimation methods use traditional physics-based models or modern data-driven machine learning techniques. Tires have a complex structural and material makeup, which makes it challenging to characterize their mechanical properties^30^. Therefore, developing theoretical models is crucial for enhancing this understanding. These models are generally based on detailed studies of the tire’s physical prototype and deformation mechanisms, from which mathematical descriptions of the mechanical behaviour can be derived. Among these models, the FRTMs introduced by Gong^7^ have been widely used to study tire natural frequencies and vibration transmission across a broad frequency range (0–300 Hz). A key benefit of these models is their ability to provide closed-form expressions that describe circumferential strain patterns, making them well-suited for evaluating carcass deformation. However, their primary drawback is the coupling between deformation equations and motion equations.

**Fig. 2:**
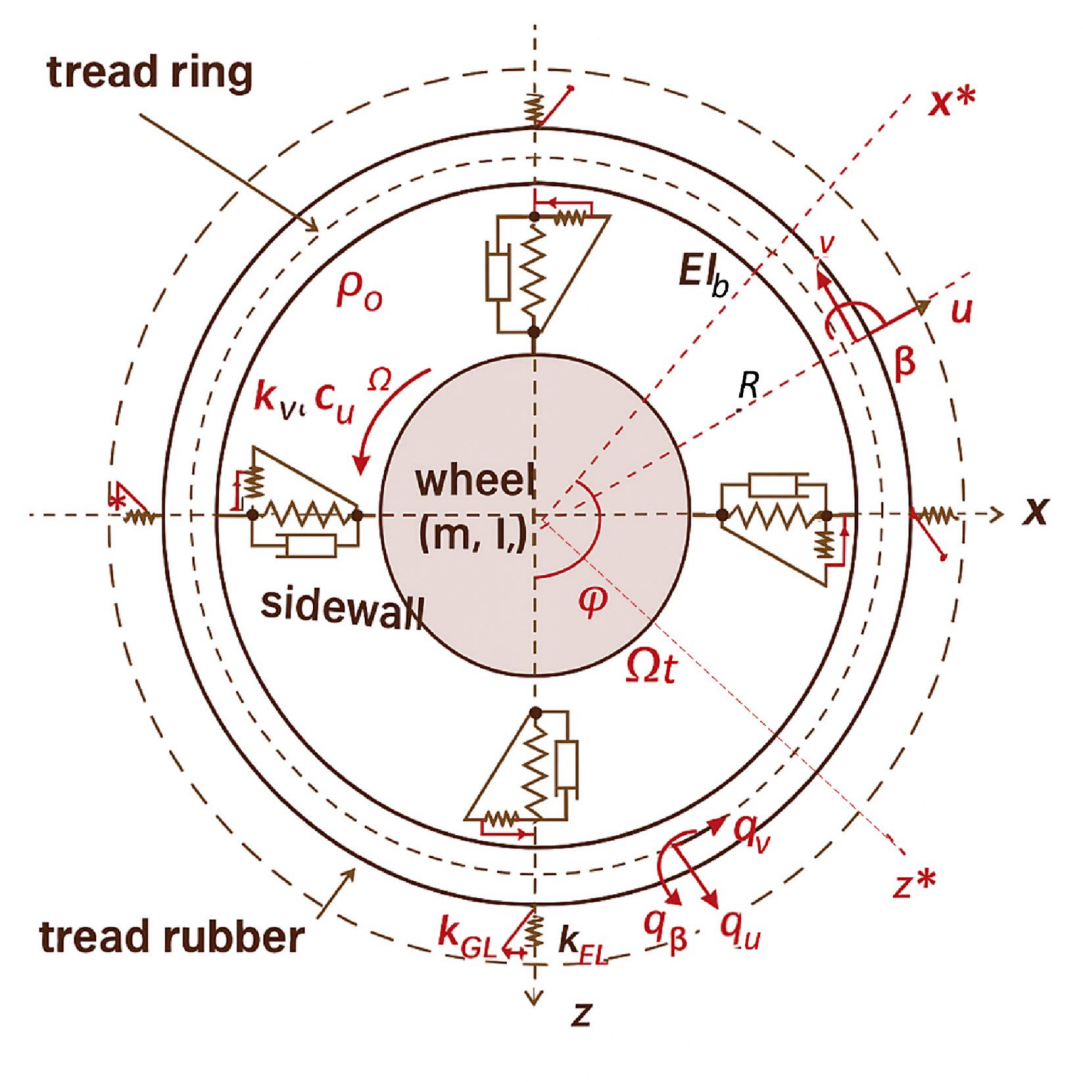
Flexible ring tire models of the tire-wheel and coordinate systems.

This work investigates steady-state deformation of the tire carcass under applied loads is analyzed to obtain a closed-form solution for tire deflection. The tread band is modeled as a homogeneous, thin, circular elastic ring supported internally, both circumferentially and radially, by a viscoelastic foundation. As shown in Fig. 2, this support is represented by spring-damper elements in the tangential and radial directions, capturing the combined effects of sidewall stiffness and the tensile stiffness of the pressurized toroidal carcass^7^.

Where $\:u\left(\theta\:\right),\:v\left(\theta\:\right)$ are radial and tangential displacement of the ring, respectively, while *b* is the ring width and *h* represents the ring thickness. *A* denotes the ring cross-section (*A* = *bh*), *E* refers to the ring Young’s modulus of the material, and *I* is the inertia moment of the cross-section of the ring. *R* represents a radius of the ring (tire treadband), while *ρ* is the density of the material.

$\:{{k}_{w},\:k}_{v}$ are stiffness per unit length in radial and tangential directions, respectively. While $\:{k}_{x}$ represents the stiffness of the tangential bristle. $\:{\rho\:}_{0}$ is inflation pressure, *Ω* denotes wheel rotation speed peed and *ε*_*x*_ refers to slip ratio. *Φ*, *θ* are rotational angles in inertial and rotating frames, respectively. While $\:{c}_{w}$, $\:{c}_{v}\:$are damping per unit length in radial direction and tangential direction, respectively.

Analyzing the contact forces and deformation using a rotating coordinate system is especially beneficial. In this system, the cylindrical coordinates (*r*, *φ*) and (*r*, *θ*) indicate the position of an infinitesimal ring element in a translational coordinate system and a rotating coordinate system, respectively. In both cases, the origin is located at the wheel center. The (*x*, *z*) and (x*, z*) coordinates correspond to the inertial reference frame, while the latter pair defines the rotating reference frame.

The two coordinate systems are related by the following equations:

1 $$\:x={x}^{*}\mathrm{cos}\left(\varOmega\:t\right)-{z}^{*}\mathrm{sin}\left(\varOmega\:t\right)$$

2 $$\:z={x}^{*}\mathrm{sin}\left(\varOmega\:t\right)-{z}^{*}\mathrm{cos}\left(\varOmega\:t\right)$$

3 $$\:\varPhi\:=\theta\:+\left(\varOmega\:t\right)\:$$

Here, *Ω* represents the wheel’s angular velocity, and *t* denotes time. The position of a small segment of the tread band ring is given in cylindrical coordinates (*R*, *φ*) relative to the fixed (non-rotating) reference frame (*x*, *z*), as depicted in Fig. 2. The variables $\:{q}_{w}\left(\theta\:,t\right)$ and $\:{q}_{v}\left(\theta\:,t\right)$ represent the external loads applied in the tangential and radial directions, respectively. Following Bernoulli–Euler beam theory, the tread band is idealized as an inextensible, curved beam primarily subjected to bending. Because of the high extensional stiffness of the modern radial tire, the middle surface of the tire carcass is assumed to be inextensible. Hence, the radial deformation *w* and tangential deformation *v* of the tire carcass in a circumferential position are governed by the following relation:

4 $$\:w\left(\theta\:,t\right)=-\frac{\partial\:v\left(\theta\:,t\right)}{\partial\:\varphi\:\left(\theta\:,t\right)}$$

The displacement fields, defined relative to any point on the mid-surface of the elastic ring, act as the main kinematic variables in the analysis. From the governing motion equations for both the treadband and the wheel assembly, these displacement fields, along with the radial and tangential force components, $\:{F}_{x}^{*}$ and $\:{F}_{z}^{*}$, applied at the wheel rim center, it can be represented as follows:

5 $$\:{F}_{x}^{*}=R{\int\:}_{0}^{2\pi\:}({k}_{w}wsin\left(\theta\:\right)+{k}_{v}wcos\left(\theta\:\right))d\theta\:$$

6 $$\:{F}_{z}^{*}=-R{\int\:}_{0}^{2\pi\:}({k}_{w}wcos\left(\theta\:\right)-{k}_{v}wsin\left(\theta\:\right))d\theta\:$$

The quantities $\:v\left(\theta\:\right)$ and $\:w\left(\theta\:\right)$ are described within the rotating co-ordinate system using the Modal Expansion Method (MEM), as detailed in references^31,32^:

7 $$\:v\left(\theta\:,t\right)=\sum\:_{n=1}^{+\infty\:}[{a}_{n}tcos\left(n\theta\:\right)+\:{b}_{n}\left(t\right)sin\left(n\theta\:\right)]$$

8 $$\:w\left(\theta\:,t\right)=-\frac{ǝv}{ǝ\theta\:}\sum\:_{n=1}^{+\infty\:}[n{a}_{n}tsin\left(n\theta\:\right)-\:n{b}_{n}\left(t\right)cos\left(n\theta\:\right)]$$

Here, $\:{a}_{n}\left(t\right)$ and $\:{b}_{n}\left(t\right)$ are the general model displacements.

The external line forces acting on the ring are defined by the following equations:

9 $$\:{q}_{w}\left(\theta\:,t\right)={Q}_{w}\delta\:\left(\varnothing\:-{\varnothing\:}_{0}\right)={Q}_{w}\delta\:\left(\theta\:-{\theta\:}_{0}\right)={Q}_{w}\delta\:\left(\theta\:-{(\varnothing\:}_{0}-\varOmega\:t\right))$$

10 $$\:{q}_{v}\left(\theta\:,t\right)={Q}_{v}\delta\:\left(\varnothing\:-{\varnothing\:}_{0}\right)={Q}_{v}\delta\:\left(\theta\:-{\theta\:}_{0}\right)={Q}_{v}\delta\:\left(\theta\:-{(\varnothing\:}_{0}-\varOmega\:t\right))$$

In these, δ represents the Dirac delta function, while $\:{Q}_{w}$ and $\:{Q}_{v}$ denote the magnitudes of radial and tangential line forces, respectively, referenced to a fixed angular position $\:{\varnothing\:}_{0}$ in the stationary coordinate frame. Under steady-state operating conditions, the tangential displacement of the tire tread band and the corresponding radial displacement caused by these concentrated line forces can be determined by applying the Modal Expansion Method (MEM):

11 $$\:v\left(\varPhi\:\right)=\sum\:_{n=0}^{+\infty\:}[{A}_{n1}{Q}_{v}cosn\left({\varnothing\:}_{0}-\varnothing\:+{\gamma\:}_{n}\right)+{A}_{n2}{Q}_{w}sinn\left({\varnothing\:}_{0}-\varnothing\:+{\gamma\:}_{n}\right)\:]$$

In this expression, $\:{A}_{n1}$, $\:{A}_{n2}$, and $\:{\gamma\:}_{n}$ are model parameters influenced by the initial stress in the tread band, which arises from centrifugal loading and tire inflation pressure. This initial stress depends on the wheel’s angular velocity *Ω* and the inflation pressure $\:{\rho\:}_{0}$, as given by the following relation:

12 $$\:{\sigma\:}_{\theta\:\:}^{0}A=({\rho\:}_{0}bR+\:\rho\:A{R}^{2}{\varOmega\:}^{2})$$

When a distributed load is applied over the tire–road contact patch, bounded by the front and rear angular positions *Φ*(*f*) and *Φ*(*r*), respectively, the FRTM solution is estimated through the principle of superposition. Using this principle, the resulting radial and tangential pressure distributions, $\:{q}_{w}$and $\:{q}_{v}$, assuming a symmetric contact patch of extent *Φ*(*r*)-*Φ*(*f*), can be expressed as:

13 $$\:{q}_{w}\left(\varPhi\:\right)=\frac{3}{4}\frac{{F}_{z}({\varPhi\:}_{r}^{2}-{\varPhi\:}^{2})}{R{\varPhi\:}_{r}^{3}cos\left(\varPhi\:\right)}\:+\:{\tau\:}_{x}\left(\varnothing\:\right)sin\left(\varPhi\:\right)$$

14 $$\:{q}_{v}\left(\varPhi\:\right)={\tau\:}_{x}\left(\varnothing\:\right)cos\left(\varPhi\:\right)$$

Here, $\:{F}_{z}$ represents the vertical load applied to the wheel, while $\:{\tau\:}_{x}$represents the tangential force per unit length along the contact patch. The evaluation of $\:{\tau\:}_{x}$ is performed using a generalized Coulomb friction model, formulated as:

15 $$\:{\tau\:}_{x}=\:\left\{\begin{array}{c}\left({\varnothing\:}_{f}-\varnothing\:\right){k}_{x}{\epsilon\:}_{x}\:,\:\:\left({\varnothing\:}_{f}-\varnothing\:\right){k}_{x}{\epsilon\:}_{x}\:\le\:{\mu\:}_{s}{q}_{z}\left(\varnothing\:\right)\:\:\\\:-\:{\mu\:}_{d}{q}_{z}\left(\varnothing\:\right)sign\left(\:{\epsilon\:}_{x}\right),\:\:\:\:\:\:\:\:\:\:\:\:\:\:elesewhere\end{array}\right.$$

In this equation, $\:{q}_{z}\left(\varnothing\:\right)$ denotes the estimated normal pressure distribution over the contact patch, $\:{\epsilon\:}_{x}$ is the slip ratio, and $\:{k}_{x}$ represents the bristle stiffness parameter. The terms $\:{\mu\:}_{d}$ and $\:{\mu\:}_{s}$ correspond to the dynamic and static friction parameters, respectively.

By dividing the contact patch $\:{N}_{\varPhi\:}$ into equal angular segments of size Δ$\:\varnothing\:$, the resulting concentrated forces $\:{Q}_{w,i}$ and $\:{Q}_{v,i}$ acting at a point with angular coordinate $\:{\varPhi\:}_{0,\:\:},\:i$ is expressed as:

16 $$\:{Q}_{w,i}=\:{q}_{w}\left({\varPhi\:}_{0,\:\:},\:i\:\right)\varDelta\:\varnothing\:\:\:\:$$

17 $$\:{Q}_{v,i}=\:{q}_{v}\left({\varPhi\:}_{0,\:\:},\:i\:\right)\varDelta\:\varnothing\:\:$$

Therefore, by superimposing the individual solution components, the resulting tangential and radial displacement fields can be written as:

18 $$\:v\left(\varPhi\:\right)=\sum\:_{i=1}^{{N}_{\varPhi\:}}\sum\:_{n=0}^{+\infty\:}[{A}_{n1}{Q}_{v,i\:}\mathrm{c}\mathrm{o}\mathrm{s}(n\left({\varnothing\:}_{0,i}-\varnothing\:+{\gamma\:}_{n}\right))+{A}_{n2}{Q}_{w,i}\mathrm{s}\mathrm{i}\mathrm{n}(n\left({\varnothing\:}_{0,i}-\varnothing\:+{\gamma\:}_{n}\right)\left)\:\right]$$

19 $$\:w\left(\varPhi\:\right)=\sum\:_{i=1}^{{N}_{\varPhi\:}}\sum\:_{n=0}^{+\infty\:}n[-{A}_{n1}{Q}_{v,i\:}\mathrm{s}\mathrm{i}\mathrm{n}(n\left({\varnothing\:}_{0,i}-\varnothing\:+{\gamma\:}_{n}\right))+{A}_{n2}{Q}_{w,i}\mathrm{c}\mathrm{o}\mathrm{s}(n\left({\varnothing\:}_{0,i}-\varnothing\:+{\gamma\:}_{n}\right)\left)\:\right]$$

Thus, by combining these components, the tangential and radial displacement fields are expressed as^31,32^:

20 $$\:{\epsilon\:}_{\theta\:}=\frac{y}{{R}^{2}}(\frac{\partial\:v}{\partial\:\theta\:}-\frac{{\partial\:}^{2}v}{{\partial\:\theta\:}^{2}})$$

and by substituting in the formulas of $\:v\left(\theta\:\right)$ and $\:w\left(\theta\:\right)$.

21 $$\:{\epsilon\:}_{\theta\:}\left(\varPhi\:\right)=\sum\:_{i=1}^{{N}_{\varPhi\:}}\sum\:_{n=0}^{+\infty\:}n[-{A}_{n1}{Q}_{v,i\:}\mathrm{s}\mathrm{i}\mathrm{n}(n\left({\varnothing\:}_{0,i}-\varnothing\:+{\gamma\:}_{n}\right))-{A}_{n2}{Q}_{w,i}\mathrm{c}\mathrm{o}\mathrm{s}(n\left({\varnothing\:}_{0,i}-\varnothing\:+{\gamma\:}_{n}\right)\left)\:\right]$$

Although the model is primarily based on strain analysis, its practical application requires integrating additional sensing methods. Specifically, because the model accounts for the initial tire inflation pressure, a tire pressure sensor is necessary to supply this input parameter. Moreover, in real-world scenarios, measuring tire load involves using force sensors, usually installed on the vehicle chassis, to work in conjunction with strain measurements.

In this study, a two-dimensional ring model is created to examine the dynamic response within the wheel plane. This work introduces a coupled model that integrates both rigid and flexible rings, facilitating the representation of tire elastic deformation while ensuring a rapid solution process for transient dynamic analysis. The coupled rigid-flexible ring model consists primarily of three elements: a flexible ring, a rigid ring, and a damped spring element that is a simplified representation of the tire’s sidewall. Initially, the flexible ring model is employed to theoretically investigate the deformation and contact characteristics of the tire, allowing for the determination of the tire’s steady-state deformation and the forces acting on the contact patch. Concurrently, the rigid ring model is utilized to address the overall motion and dynamic response of the tire. Ultimately, the interrelationship between these two ring models is established, and a comprehensive theoretical expression along with the calculation process for the coupled rigid-flexible ring model is provided, establishing a theoretical basis for tire dynamics analysis.

The equations governing the rigid and flexible rings are integrated to form a complete computational model. The contact force derived from the local deformations in the flexible ring model is treated as the excitation for the rigid ring. Once the velocity and coordinates of the rigid ring are determined through dynamic analysis, the simulation procedure is carried out.

The equation of motion for a coupled model that integrates both rigid and flexible rings is expressed as

22 $$\:{M}_{n}{\ddot{a}}_{n}+\:{C}_{n}{\dot{a}}_{n}+{G}_{n}{\dot{a}}_{n}+{K}_{n}{a}_{n}={F}_{n}$$

$$\:{M}_{n}=\:\left[\begin{array}{cc}{m}_{n}&\:0\\\:0&\:{m}_{n}\end{array}\right],\:\:{C}_{n}+{G}_{n}=\:\left[\begin{array}{cc}{c}_{n}&\:{g}_{n}\\\:{-g}_{n}&\:{c}_{n}\end{array}\right],\:{K}_{n}=\:\left[\begin{array}{cc}{k}_{n}&\:0\\\:0&\:{k}_{n}\end{array}\right],\:\:{a}_{n}=\left[\begin{array}{c}{a}_{1\mathbf{n}}\\\:{a}_{2\mathbf{n}}\end{array}\right],\:\:{F}_{n}=\left[\begin{array}{c}{F}_{\mathrm{n}1}\\\:{F}_{\mathrm{n}2}\end{array}\right]\:$$.

The coefficients are listed as

23 $$\text{Modal equivalent mass}\:{m}_{n}=\rho\:A\left(1+{n}^{2}\right)$$

24 $$\text{Modal damping factor }\:{G}_{n}=\left(n\varOmega\:\right){g}_{n}\; \mathrm{and} \; {C}_{n}=\left(n\varOmega\:\right){c}_{n}$$

Modal equivalent Stiffness coefficient

25 $$\:{k}_{n}=\left(\frac{EI}{{R}^{4}}{n}^{2}+\frac{{\sigma\:}_{\theta\:}^{0}}{{R}^{2}}\right){\left(1-{n}^{2}\right)}^{2}-\frac{{P}_{0}b}{R}\left(1-{n}^{2}\right)+{k}_{v}+{k}_{w}{n}^{2}-\rho\:A\left(1+{n}^{2}\right){\varOmega\:}^{2}$$

As a result, the model is inherently and closely linked to the use of these supplementary sensing systems. The related model parameters are defined as follows:

26 $$\:{A}_{n1}=\frac{1}{\pi\:\sqrt{{\left({M}_{n}-{G}_{n}\right)}^{2}+{\left({C}_{n}\right)}^{2}}}$$

27 $$\:{A}_{n2}=n{A}_{n1}$$

28 $$\:n{\gamma\:}_{n}={\mathrm{t}\mathrm{a}\mathrm{n}}^{-1}\left(\frac{{C}_{n}}{{M}_{n}-{G}_{n}}\right)$$

29 $$\:{M}_{n}={k}_{n}-{\left(n\varOmega\:\right)}^{2}{m}_{n}$$

30 $$\:{G}_{n}=\left(n\varOmega\:\right){g}_{n}$$

31 $$\:{C}_{n}=\left(n\varOmega\:\right){c}_{n}$$

32 $$\:{m}_{n}=\rho\:A\left(1+{n}^{2}\right)$$

33 $$\:{g}_{n}=-4\rho\:An\varOmega\:$$

34 $$\:{c}_{n}={c}_{v}+{c}_{w}{n}^{2}$$

35 $$\:{k}_{n}=\left(\frac{EI}{{R}^{4}}{n}^{2}+\frac{{\sigma\:}_{\theta\:}^{0}}{{R}^{2}}\right){\left(1-{n}^{2}\right)}^{2}-\frac{{P}_{0}b}{R}\left(1-{n}^{2}\right)+{k}_{v}+{k}_{w}{n}^{2}-\rho\:A\left(1+{n}^{2}\right){\varOmega\:}^{2}$$

36 $$\:{\sigma\:}_{\theta\:}^{0}A=\left({p}_{0}bR+\rho\:A{R}^{2}{\varOmega\:}^{2}\right)$$

The FRTM offers a precise computational representation of circumferential strain, making it a useful tool for monitoring and diagnostic purposes in intelligent tire systems. Table 1 presents the parameters in the intelligent tire technology that influence sensor outputs.

**Table 1:** Parameters in intelligent tire technology that influence sensor outputs.

Category	Parameter
Tire condition and performance	Pressure, temperature, wear, and rotation speed
Tire–road interaction	Longitudinal force, *F*_*x*_, Lateral force, *F*_*y*_, Vertical force, *F*_*z*_, aligning moment, *M*_*z*_, contact patch length, Slip ratio, Slip angle
Road condition	Friction coefficient and road surface classification
Vehicle motion and driving behaviors	Vehicle velocity, acceleration, and braking turning

The equations derived encompass sensor output, the relationship between radial and tangential displacement, circumferential strain, external line forces, steady-state response for concentrated loads, response to distributed loads, normal pressure distribution, tangential force per unit length, radial and tangential forces per unit length, and a discrete approach for concentrated loads. The tire model proposed is capable of estimating tire deformations with a satisfactory level of precision across various operational working conditions.

As the number of tire sensor technologies expands, including inflation pressure monitoring and acceleration sensors, the tire itself evolves into a sensor. This supplementary data can be leveraged to enhance the efficiency of vehicle dynamics controllers (VDC), advanced driver assistance systems (ADAS), and numerous automated driving functions, thereby promoting electro-mobility.

## Simulation results of smart tire strain behaviour

This section presents a complete simulation analysis using the developed mathematical framework for modelling tire forces and moments. The goal is to evaluate whether the proposed theoretical model meets the desired performance criteria regarding tire dynamic behaviour, with a particular focus on using strain gauge sensors to measure tire deformation. An initial set of simulations was conducted to analyse the qualitative behaviour of both displacement and strain, using parameter values reported in^28,33,34^ and summarized in Table 2.

**Table 2:** Simulations parameters used for flexible ring tire models (FRTM).

Parameters	b	h	E_I_	*R*	ρ_A_	K_w_	K_v_	C_w_ = C_v_	K_x_	*P*_0_
Value	0.14	0.01	2.0	0.3	3.15	6.3 × 10^5^	6.3 × 10^5^	0	5.43 × 10^5^	1.2 × 10^5^
Unit	*m*	*m*	*Nm²*	*m*	*Kg/m*	*N/m²*	*N/m²*	*N/s m²*	*N/m*	*N/m²*

**Fig. 3:**
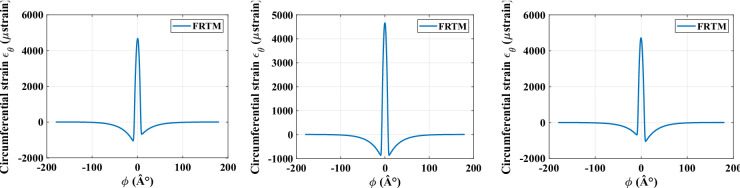
Comparison between circumferential strains computed with different numbers of modes: (**a**), (**b**), and (**c**) at n_mode = 10; (**d**), (**e**), and (**f**) at n_mode = 100.

Figure 3 shows a comparison of circumferential strain distributions obtained through the modal expansion method, using ten modes (n_mode = 10, Fig. 3a–c) and one hundred modes (n_mode = 100, Fig. 3d–f). In both cases, a radial load of 1000 *N* is applied with zero longitudinal slip. The simulation results indicate that the tire’s circumference reaches its maximum strain at the center of the contact patch.

**Fig. 4:**
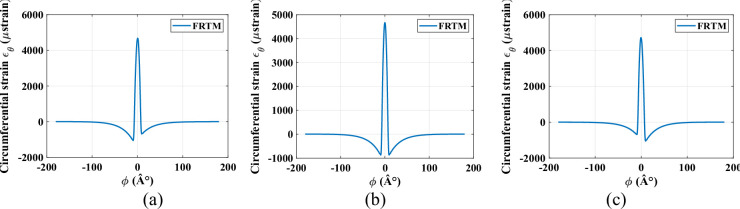
Strain behavior for different values of the longitudinal slip: (**a**) *ε*_*x*_= − 0.2; (**b**) *ε*_*x*_ = 0, (**c**) *ε*_*x*_ = 0.2.

Using a 100-mode expansion improves the accuracy of circumferential strain predictions by effectively removing oscillations at the lower peak values. The peak circumferential strain magnitude is directly affected by the vertical load applied to the rim. Figure 4 further demonstrates the FRTM’s ability to replicate circumferential strain behaviour under different longitudinal slip conditions (*ε*_*x*_ = {− 0.2, 0, 0.2}). The results reveal asymmetry in the lower peak regions, with simulations conducted under a constant radial load of 250 *N*.

**Fig. 5:**
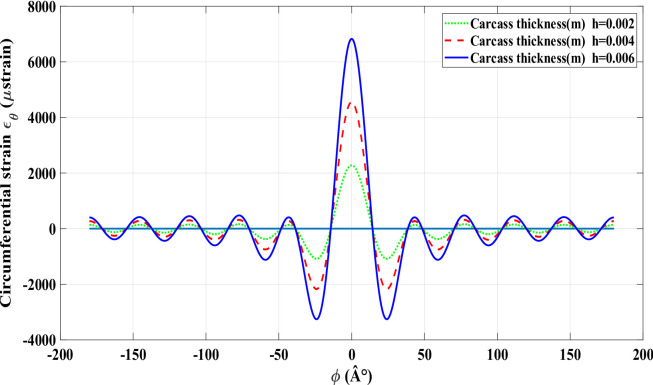
Strain signal curves under different values of wear with a number of modes n_mode = 10.

**Fig. 6:**
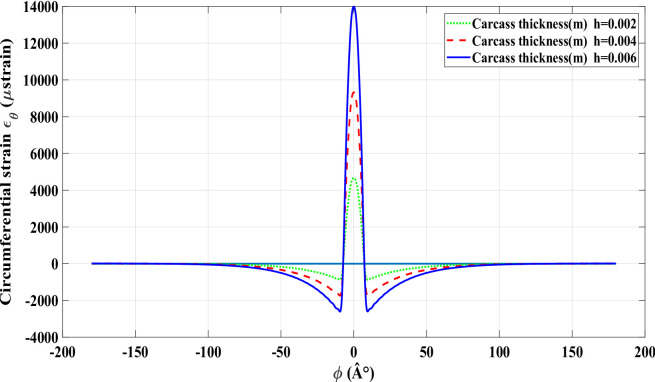
Strain signal curves under different values of wear with a number of modes n_mode = 100.

An increase in the applied radial load leads to a corresponding rise in the central peak value of the circumferential strain, as shown in Fig. 4 for a radial load of 1000 *N* with zero longitudinal slip (*ε*_*x*_ = 0). The tire strain behaviour, modelled using the FRTM, validates the model’s effectiveness for intelligent tire monitoring applications. Accurately replicating the circumferential strain profile in smart tires lays the groundwork for developing estimation tools that can infer tire–road contact forces, thereby improving vehicle performance, driving safety, and passenger protection. Figures 5 and 6 display the strain signals predicted by the refined physical model. Comparing these plots reveals significant changes in the strain pattern as tire wear advances. The main impact of wear on the strain response is seen at the trailing edge of contact patch, where the difference between trailing-edge and leading-edge strain magnitudes becomes more distinct. It is important to note that during rolling, every measurement point on the tire experiences deformation upon entering the contact area, inherently producing tensile strain. Automobile and tire manufacturers are innovating technologies such as the smart strain sensor to directly assess these variations. By employing sensors within the tire to track dynamic strain, a proprietary algorithm is capable of estimating the tire’s wear status. As the tire experiences wear and aging, its internal strain signature transforms, indicating the continuous alterations in its physical characteristics and road-contact dynamics. As shown in Figs. 5 and 6, the strain signal curves under different values of wear with a number of models.

**Fig. 7:**
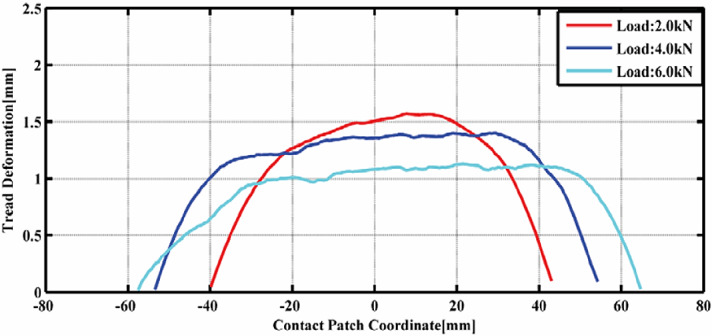
Tread deformation under different values of loads.

Furthermore, simulations are conducted under three different loads to examine the effect of wheel load on tire-tread deformation, as shown in Fig. 7. The findings from these simulations indicate that a higher load results in a decrease in tread deformation, which can be explained by the transfer of load from the tire crown to the tire shoulder. Nevertheless, it is also observed that the deformation patterns become progressively asymmetric as the load increases. This finding is significant for a comprehensive understanding of the tire rolling resistance mechanism, as it underscores the differences in deformation under varying load conditions. An increased load leads to a greater rolling resistance force, which is consistent with both common experiences and experimental data suggesting that rolling resistance is directly proportional to wheel load within a defined operational range.

The proposed mathematical model has significant engineering applications, including its use in real-time tire condition monitoring, tire wear prediction, and structural optimization for enhanced performance and safety. Furthermore, the model’s integration with vehicle control systems—such as anti-lock braking (ABS), traction control, and stability management—can enable adaptive, data-driven control strategies that respond dynamically to changes in tire–road interactions. This integration underscores the model’s practical relevance, offering a foundation for intelligent mobility solutions in next-generation electric and autonomous vehicles.

Table 3 illustrates the difference between the proposed mathematical model and traditional mathematical models.

**Table 3:** The proposed mathematical model versus traditional mathematical models.

Feature	Traditional mathematical models	The proposed mathematical model
Complexity	Primarily empirical, based on fitting mathematical equations to experimental data	Physically based, incorporating more realistic tire construction and material properties
Physical representation	Simplified representation, often treating the tire as a “black box” that relates input (slip angle, load) to output (forces, moments). The Brush model is an early analytical example that simplifies the contact patch	Detailed representation of the tire’s structure, including the carcass, belt, and tread. This allows for the simulation of complex interactions
Accuracy	Accurate within the specific, controlled conditions (e.g., speed, load, friction) under which the experimental data was collected	Valid across a much wider range of operating conditions, including scenarios beyond typical testing parameters
Handling of dynamics	Limited ability to model unsteady characteristics or non-linear relationships	Better at modeling dynamic and unsteady characteristics, including variations in load and slip
Computational cost	Generally, lower computational time	Higher computational time due to increased complexity and numerical methods

## Conclusions and future work

In this paper, a new coupled rigid-flexible ring model has been introduced to examine the dynamic response characteristics of tires. The contact algorithms, which utilize a 2D flexible ring, facilitate the analysis of pressure distribution within the tire-road contact area under varying vertical loads. This study reviews recent progress in intelligent tire technologies, focusing on the feasibility of characterizing tire behaviour through strain-based sensing methods. It highlights both contact-type and non-contact-type commercially available sensors capable of estimating parameters related to tire–road interaction. Additionally, an analytical formulation of the FRTM has been developed, and its application to strain-based smart tire systems is investigated through numerical simulations. These simulations assess tire characteristics and physical quantities across various model mode numbers. Closed-form solutions have also been derived for tire carcass displacements and circumferential strain fields. Moreover, an investigation into strain signal curves across different wear values and several modes was incorporated. Furthermore, simulations were conducted under three separate loads to assess the impact of wheel load on tire tread deformation. Notably, the impact of tire wear on the resulting strain signals has been examined, revealing distinctive patterns that could serve as reliable indicators for evaluating or predicting tire wear during operation. This feature adds a substantial amount of value. Predictive maintenance and better tire management are made possible by these improved features. In the future work, one could conduct real-world testing with instrumented tires (e.g., embedded strain gauges, accelerometers) to validate the FRTM’s predictions against empirical data. Additionally, one could combine the physics-based FRTM with machine learning and neural networks to refine predictions using real-world sensor data.

## Data Availability

No datasets were generated or analysed during the current study.
